# Choice modelling with Gaussian processes in the social sciences: A case study of neighbourhood choice in Stockholm

**DOI:** 10.1371/journal.pone.0206687

**Published:** 2018-11-05

**Authors:** Richard P. Mann, Viktoria Spaiser, Lina Hedman, David J. T. Sumpter

**Affiliations:** 1 Department of Statistics, School of Mathematics, University of Leeds, Leeds, United Kingdom; 2 The Alan Turing Institute, London, United Kingdom; 3 School of Politics and International Studies, University of Leeds, Leeds, United Kingdom; 4 Institute for Housing and Urban Research, Uppsala University, Uppsala, Sweden; 5 OTB – Research for the Built Environment, Faculty of Architecture and the Built Environment, Delft University of Technology, Delft, The Netherlands; 6 Department of Mathematics, Uppsala University, Uppsala, Sweden; The University of Memphis, UNITED STATES

## Abstract

We present a non-parametric extension of the conditional logit model, using Gaussian process priors. The conditional logit model is used in quantitative social science for inferring interaction effects between personal features and choice characteristics from observations of individual multinomial decisions, such as where to live, which car to buy or which school to choose. The classic, parametric model presupposes a latent utility function that is a linear combination of choice characteristics and their interactions with personal features. This imposes strong and unrealistic constraints on the form of individuals’ preferences. Extensions using non-linear basis functions derived from the original features can ameliorate this problem but at the cost of high model complexity and increased reliance on the user in model specification. In this paper we develop a non-parametric conditional logit model based on Gaussian process logit models. We demonstrate its application on housing choice data from over 50,000 moving households from the Stockholm area over a two year period to reveal complex homophilic patterns in income, ethnicity and parental status.

## Introduction

People’s choices depend on their personal characteristics, their socio-economic status and their aspirations. When those choices are connected to socioeconomic indicators such as income, wealth and ethnicity they can aggregate into profoundly important emergent social phenomena such as segregated neighbourhoods, schools and workplaces. It is vital therefore to be able to accurately determine, at the individual level, the factors influencing socially relevant choices. The data revolution in social science promises to transform our ability to learn about individual behaviour in high powered quantitative studies. To fully realise the power of large data sets requires models that are flexible enough to accommodate many different social phenomena while also being statistically robust. These models should place interpretability at their core, while leveraging modern computational power and abundant data to reveal patterns of behaviour that can escape simpler models and smaller experimental studies. In this paper we will propose a new development of a standard statistical methodology, the conditional logit model, to answer these needs.

Conditional logit models are a class of multinomial, discrete choice models first proposed by McFadden [1, 2]. Discrete choice models specify behaviour in which individuals choose one option from a set of given alternatives C. The discrete choice model approach deviates from classical regression-type models since the dependent variable is not a quantitative measure of some outcome, but rather an indicator of whether or not some outcome (choice) has occurred. Therefore, discrete choice models make probabilistic statements about the occurrence of certain events, i.e. choices; they model probabilities of events rather than conditional mean functions [3, 4]. Multinomial choice models are those that deal with more than two possible choice options, which typically do not follow a natural ordering. Based in econometrics logic, it is typically assumed that from a set of given alternatives C, individuals select the option with the greatest utility to them. For discussions of alternatives to utility maximisation models, such as random regret models, see [5, 6].

Multinomial choice models are widely used to model consumer choices such as brands, transport options, service option, energy suppliers etc. [3, 7–10], political party choices in elections [11, 12], demographic choices like the choice of dating or marriage partners or the choice of cohabiting forms [13], to model neighbourhood selection [2, 14–17] or school selection [18]. Essentially, people make choices every day and understanding people’s choice making allows us to make predictions on people’s future choices, which again can be useful for policy makers to design better policy measures or for business to create products and services that people want.

There are many variants of multinomial choice models [3, 7]. A key element in model specification is whether the analysis focuses on the characteristics of the individuals making the choice, the characteristics of the choices themselves, or both. Conditional logit models are the canonical example of this third category, which describe the probability of choosing an option as a function of choice characteristics, that may or may not interact with individual characteristics [3, 4]. Such models can be specified in various ways, but fundamentally they follow some basic principles. The propensity to choose one of the options is assumed to be driven by a latent function, which represents which choice attributes are valued by individuals and possibly how individual characteristics may have an impact on what attributes are valued by the respective individuals. This function is often interpreted as an indirect estimate of utility. The latent function $U_{ij}^{*}$ of choice *j* to individual *i* is represented as a function of the observed individual characteristics, *X*_*i*_, the observed choice characteristics *Z*_*j*_ and unobserved attributes of both the choice and individual, *ϕ*_*ij*_:

$$U_{ij}^{*}=f\left(X_{i},Z_{j}\right)+\phi_{ij}$$ (1)

The individual *i* chooses option *j* if it offers the greatest utility, thus, the choice depends on the difference of utility between various options. This utility comparison takes place within individuals and therefore factors that influence the level of utility for all alternatives in the same way, such as individual characteristics, which are constant within individuals, can not explain an individual’s decision, they would cancel in choice probability. Individual characteristics start playing a role when they interact with alternative characteristics, i.e. according to [14]

$$U_{ij}^{*}=\beta Z_{j}+\gamma Z_{j}X_{i}+\phi_{ij},$$ (2)

where *β* and *γ* are vectors of coefficients representing the importance of the choice characteristics and interaction effects respectively.

Conditional logit models as other discrete choice models typically assume that the unobserved term follows a type I extreme value (Gumbel) distribution: *p*(*ϕ*_*ij*_) = exp(− exp(*ϕ*_*ij*_)). Independence of this term is assumed across all choices and individuals, moreover identical variances and same parameters for all individuals [3, 4]. The resulting probability function is then

$$\begin{array}{c} \begin{array}{cc} P(\text{choice}=j|X_{i},Z_{j}) & =P(U_{ij}^{*}>U_{ik}^{*},\;\forall k\in C,k\neq j)\\ & =\frac{\text{exp}(\beta Z_{j}+\gamma Z_{j}X_{i})}{\sum_{k\in C}\;\text{exp}\left(\beta Z_{k}+\gamma Z_{k}X_{i}\right)} \end{array} \end{array}$$ (3)

The summation is over all possible choices in the set of possible options, C. The specific form of the likelihood function results from assuming that individuals optimise their true utility, including the unseen component (e.g. use of normally-distributed unseen components would result in a multinomial probit model). Alternatively, ignoring the random component, the logistic probability may be viewed as an expression of individual’s limited rationality in the light of the observed characteristics; individuals are most likely to choose the option with greatest utility, but may also make suboptimal choices of options with relatively high but non-maximal utility (see e.g. [19, 20] for application of this principle in animal behaviour). The model parameters, and thus the utility function, is typically estimated using maximum likelihood estimation (MLE) [3, 4].

The linearity of the latent (utility) function in classic multinomial choice models limits the range of individual preferences that can be inferred to monotonically increasing or decreasing functions of the option characteristics. But, the individuals preference on *Z* may be non-monotonic and/or highly non-linear. Indeed, attempts have been made to extend the linear utility function by using for instance polynomial combinations of *X* and *Z*. For example, Bruch & Mare [15] used a quadratic form to estimate the utility of neighbourhoods based on the percentage of own/group residents. This can allow for more sharply increasing or decreasing utilities around thresholds or non-monotonic utility functions. Combined with step functions and staircase functions as employed by [15] this can capture more complex and realistic preferences, but the types of behaviour that such a utility can model depends on the explicit choice of basis functions used. For example, a quadratic form will not be able to model multiple-modal preferences, and may underestimate particularly sharp transitions in the utility function. Other non-linear approaches to multinomial choice modelling were suggested by [21–24]. However, all these were approaches offering only highly specific modes or specific modifications and tweaks for specific problems, not a general alternative approach to the standard linear random utility maximisation problem.

Here we take a very different approach. We tackle the problem of model inflexibility by utilising techniques primarily addressed in machine-learning that are optimised for learning from large data sets with modern computational power. Following the work of [25] introducing Bayesian inference for logistic Gaussian process density estimation, we suggest a non-parametric conditional logit model, based on Gaussian processes, to allow for a large variety of complex preferences that vary between individuals without a combinatorial explosion of parametric basis functions. Instead of postulating a parametrised utility function and testing its fit to the data, we derive the form of the utility function directly from the data. The model exhibits flexibility, being able to infer any continuous and relatively smooth utility function. We further show that this model is statistically robust and automatically determines the relevance of putative predictive factors. It provides a natural framework for model selection, and therefore formal feature selection.

Hence, we merge an econometric model, the conditional logit multinomial choice model, with a powerful and highly flexible machine learning method, Gaussian process logit models [25, 26]. To demonstrate this methodology we analyse a unique data set of over 50,000 neighbourhood choices by households in the Stockholm economic area, inferring a complex utility functions based on household income, ethnicity, age and number of children; and on neighbourhood mean income, percentage of non-western residents, percentage of households with children and distance from the current abode. The structure is as follows. First we introduce Gaussian processes, specify the Gaussian process conditional logit model and derive the learning algorithm for inference of the utility function. We show how the standard linear model can be retrieved as a special case of our extended model.

Secondly we specify a precise implementation of our model for the case of household neighbourhood selection, and perform inference to reveal the utility function linking individuals’ characteristics to their neighbourhood preferences. In doing this we quantitatively test which of two models gives the best description of the data: (i) the classic linear model; (ii) our non-parametric model. We also identify which of the neighbourhood characteristics in the data are predictive of neighbourhood choice. Finally we discuss the form of the revealed utility function, with emphasis on homophilic preferences, and detail the methodological advances made alongside remaining limitations.

## 1 Methodology: The Gaussian process conditional logit model

In principle, the utility can be an arbitrary function, *f*(⋅) of the individual and choice characteristics

$$U_{ij}=f\left(X_{i},Z_{j}\right).$$ (4)

When data on individual choices is plentiful, complex preferences can be inferred by a sufficiently flexible model. However, additional model flexibility introduces the possibility of overfitting, where finite-data effects are built into the structure of the inferred utility function. For example, a separate utility function could be derived for all individuals within each decile of personal income, allowing preferences to vary arbitrarily between these groups. However, the choices made by these 10 finite-sized groups would necessarily be different by chance, even if the fundamental neighbourhood preference did not vary with personal income. To avoid overfitting to these finite data effects we need a methodology that includes regularisation to favour simpler models. Gaussian processes are a framework for function inference that provides both flexibility in model specification, and in-built regularisation through a prior probability distribution over functions that places greater probability on smoother functions. Rasmussen and Williams [27] in particular have noted that the Gaussian process inference framework acts as an ‘automatic Occam’s razor’. Where possible we follow the notation of [27] in the following.

### 1.1 Gaussian processes

Gaussian processes (GPs) are a powerful and flexible framework for performing inference over functions [27–30]. GPs are at the core of recent development of machine-learning and have enabled an array of powerful algorithms for optimisation [31], search problems [32], change and fault detection [33], and data integration [34] among other tasks. In addition, application of these ideas to scientific questions has led to novel analyses of data-rich experiments in such fields as animal movement and navigation [35], protein sequence clustering [36] and prediction of future employment patterns [37]. Developing a Gaussian process framework for the conditional logit model potentially makes the analytical richness of these methods available to the study of sociological choice data as well. Previous theoretical work has developed the Gaussian process framework for Bayesian inference of logistic models [25], but this methodology has yet to be applied in the fields of social science. Additionally, GPs have been applied for the inference of utility and preference functions [38], but these models have not considered the interaction between individual characteristics and the features of the possible choices that lies at the core of the conditional logit model.

In a Gaussian process, the probability of a function, *f*(⋅), is specified by a mean function, *μ*(⋅) and a covariance function, *k*(⋅, ⋅) that determines the correlation between disparate locations on the function. If *f*(*x*) is a draw from a Gaussian process (denoted by GP) then any finite number of function values, *f*(***x***), evaluated at a set of inputs, ***x***, has a multi-variate Gaussian distribution (represented as standard by N),

$$\begin{array}{c} \begin{array}{cc} P(f(x)) & =GP(\mu \left(x\right),k\left(x,x^{\prime }\right))\\ \Rightarrow P(f(x)) & =N(\mu \left(x\right),k\left(x,x^{\prime }\right)). \end{array} \end{array}$$ (5)

By imposing correlations on the function via the covariance function, *k*(⋅, ⋅) the GP framework favours smoother, simpler utility functions *a priori* and requires substantive empirical evidence to infer more complex functions.

#### 1.1.1 Bayesian update rule

Bayes’ rule specifies how to update the probability of the function, *f*, in the light of new data D

$$\begin{array}{c} P\left(f|D\right)=\frac{P(D|f)P(f)}{p(D)}. \end{array}$$ (6)

For the purposes of this study our goal is to infer *f*, and we seek the value of *f* that maximises Eq 6—that is, the maximum *a posteriori* (MAP) estimate (see also [25]). We apply an expectation-maximisation iterative routine to jointly infer the optimal values of the latent function, *f*, and the covariance function *k*.

#### 1.1.2 Automatic relevance detection

GPs provide an automatic mechanism for judging the importance of different factors that may contribute to the utility function. Automatic relevance detection (ARD) [27] provides for different correlation lengths along different dimensions of the utility function by a specific parametrisation of the covariance function *k*(*x*, *x*′). We assume a general parametrised Matérn covariance form for *k*(*x*, *x*′) [28] that decays smoothly with increasing values of *r*, the effective distance between inputs,

$$r=\sqrt{\sum_{i}{\left(x_{i}-x_{i}^{\prime }\right)}^{2}/\sigma_{i}^{2}},$$ (7)

$$k\left(r\right)\equiv \lambda^{2}\left(1+\sqrt{3}r\right)\text{exp}\left(-\sqrt{3}r\right),$$ (8)

where *x*_*i*_ is the *i*th element of the multi-dimensional input *x*, *σ*_*i*_ is an adjustable length scale of variation of the function along the *i*th dimension and λ is an adjustable output scale parameter that controls the magnitude of *f*. The adjustable hyperparameters λ and *σ*_*i*_ can be specified in advance, or estimated from the data; in this paper we use type-II maximum-likelihood (also known as empirical Bayes) estimation—maximisation of the marginal likelihood, having marginalised over the unknown function *f*. The values of *σ*_*i*_, relative to the absolute magnitude of the data along this dimension, provides a direct, quantitative measure of the relevance of factor *i*. Higher values of *σ*_*i*_ indicate that the utility function changes little as factor *i* is varied, indicating that this factor is not highly relevant in determining utility. A benefit of this procedure, aside from identifying which factors are most important, is a more efficient use of data, since strong correlations imply that data for one value of factor *i* can be strongly informative about the value of *f* at other values of *i*.

#### 1.1.3 Retrieval of the linear model

It is worth noting that a Bayesian treatment of the traditional linear conditional logit model as per [2, 3, 14, 39] can be recovered within the Gaussian process framework through an alternative choice of the covariance function that imposes linear covariances. For example, if we want to propose a utility function,

$$U\left(X,Z\right)=\beta_{0}Z+\beta_{1}ZX$$ (9)

we should use a dot-product covariance function of the form,

$$k\left(\left[X_{i},Z_{j}\right],\left[X_{l},Z_{m}\right]\right)=\left[X_{i},Z_{j}X_{i}\right].{\left[X_{l},Z_{m}X_{l}\right]}^{T}.$$ (10)

Therefore we can directly compare our approach to traditional models using Bayesian model selection via the marginal likelihood of the data conditioned on the model [40]. This will allow us to establish that our model improves on standard methods for inferring preference functions.

#### 1.1.4 Expectation-maximisation routine

We apply an expectation-maximisation [41] (EM) iterative routine to jointly infer the optimal values of the latent function, *f*, and the parameters of the covariance function *k*. The details of the two repeated steps in this procedure are given below. We check for convergence of the EM algorithm via the change in covariance parameters between successive iterations.

### 1.2 Estimation of *f* ∣ *k*

For the purposes of numerical maximisation of Eq 6 we discretise the space of possible inputs to *f* to a computationally convenient sparsity (for instance, in our case study we will round household incomes to the nearest ten thousand Swedish kronor). This discretises our estimate of *f* to a finite vector ***f***. The associated covariance matrix, *K*, is calculated by applying the covariance function to all possible pairs of inputs, *i.e*. *K* = *k*(***x***, ***x***′). Our task is to find the MAP estimate $\hat{f}={\text{argmax}}_{f}\;\text{log}\;P\left(f|D,K\right)$ where,

$$\text{log}\;P\left(f|D,K\right)=\text{log}\;P\left(D|f,K\right)-\left(1/2\right)f^{T}K^{-1}f-\left(1/2\right)\text{log}\;\left|K\right|-\left(n/2\right)\text{log}\;2\pi -\text{log}\;P\left(D|K\right),$$ (11)

of which only the first 2 terms depend on ***f***. The derivative of logP(f∣D,K) with respect to the elements of ***f*** is:

$$\nabla \text{log}\;P\left(f|D,K\right)=\nabla \text{log}\;P\left(D|f,K\right)-K^{-1}f.$$ (12)

The derivatives ∇logP(D|f,K) can be calculated via the chain rule, with summation over the set of possible choices, S and over all data points, *y* in the data set D,

$$\begin{array}{c} \begin{array}{cc} \sum_{y\in D}\frac{\partial \;\text{log}\;P_{y}}{\partial f_{i}} & =\sum_{y\in D}\sum_{k\in S}\frac{\partial U_{k}}{\partial f_{i}}\frac{\partial \;\text{log}\;P_{y}}{\partial U_{k}}\\ & =\sum_{y\in D}\sum_{k\in S}(\left(\delta_{k,y}-P_{k}\right)I\left(k,i\right)), \end{array} \end{array}$$ (13)

where *δ*_*k*,*y*_ is the Kronecker delta function and *I*(*k*,*i*) is an indicator function that takes the value one if choice *k* corresponds to the function element *f*_*i*_ for that individual, and zero otherwise. Having determined the appropriate derivatives, we can apply any common maximisation algorithm to find the MAP estimate of *f*. We employ the Limited Memory Broyden—Fletcher—Goldfarb—Shanno (L-BFGS) algorithm [42] as implemented in Matlab by Mark Schmidt [43]. By numerical differentiation we can also obtain the Hessian of the log likelihood, that is the matrix of second derivatives of logP(D∣f,K):

$${W\equiv -\nabla \nabla \text{log}\;P\left(D|f,K\right)|}_{\hat{f}}.$$ (14)

### 1.3 Estimation of *k* ∣ *f*

Estimation of *k* in the Gaussian process context is equivalent to a Bayesian model selection problem. The covariance function specifies the prior probability of different types of functions that may map individual and choice characteristics to utilities. Within our assumed functional form for *k*(*x*, *x*′), the adjustable parameters represent models of varying complexity, corresponding to functions which are more or less variable.

As with all Bayesian model selection problems, we select an appropriate model by maximising the marginal likelihood of the data conditioned on the model, *i.e*. conditioned on the parameters of *k*(⋅, ⋅): {*σ*_1_, *σ*_2_, …, *σ*_*n*_, λ}, for an *n*-dimensional function, integrating over the unknown function *f*. The integral over ***f*** required is not analytically tractable, so we use Laplace’s method [25], approximating the posterior distribution of ***f*** by the Gaussian function *Q*:

$$P\left(f|D,K\right)≃Q\left(f|D,K\right)\equiv N(f;\hat{f},{\left(W+K^{-1}\right)}^{-1}).$$ (15)

From this we obtain the estimated log marginal likelihood:

$$\text{log}\;P\left(D|K\right)≃\text{log}\;Q\left(D|K\right)=\text{log}\;P\left(D|\hat{f}\right)-\frac{1}{2}{\hat{f}}^{T}K^{-1}\hat{f}-\frac{1}{2}\text{log}\;\left|B\right|$$ (16)

where $\hat{f}$ is the MAP estimate of *f* from the previous section, and *B* = *I* + *W*^1/2^ *KW*^1/2^. Taking the gradient of the approximate marginal likelihood with respect to each parameter of *k*(⋅, ⋅) (see [27]) we can maximise the marginal likelihood to obtain an estimate of *k*. The derivatives of the marginal likelihood with respect to a specified hyper parameter, *θ* are given by:

$$\frac{\partial \;\text{log}\;Q(D|K)}{\partial \theta }=\frac{1}{2}{\hat{f}}^{T}K^{-1}\frac{\partial K}{\partial \theta }K^{-1}\hat{f}-\frac{1}{2}\text{tr}({\left(W^{-1}+K\right)}^{-1}\frac{\partial K}{\partial \theta }).$$ (17)

#### 1.3.1 Feature selection and model comparison

The automatic relevance detection built into the GP framework will down weight the influence of extraneous factors in predictive use of the model. However, it can also be useful to perform formal feature selection to determine which individual or choice characteristics are relevant in determining the utility function. We take a Bayesian approach to feature selection and model comparison (see e.g. [44–46]). We evaluate the marginal likelihood of the data: the probability of the data after marginalising over the unknown utility function, as approximated in Eq 16. Inclusion of more individual or choice features increases the space of possible utility functions, which both increases the potential to find an appropriate function to fit the data but also decreases the prior probability of any specific function. To test whether differences in the number of model hyperparameters and thus model complexity is responsible for differences in the marginal log-likelihood, we also evaluate the Bayesian Information Criterion (BIC) [47] for each model, selecting the model with the lowest value of BIC.

## 2 Application to Stockholm residential data

The data we will be using to demonstrate our methodology are mobility histories data based on the housing records of households in the Stockholm Economic Area between 2006 and 2008 derived from GeoSweden, a longitudinal micro-database containing the entire Swedish population tracked from 1990 to 2008. Data were obtained in the form of anonymised population register records from the Swedish government agency Statistics Sweden (Statistiska centralbyrån), who gave ethical consent for the use of the data before providing access. This data is third-party, and must be requested directly from Statistics Sweden. Instructions for requesting data access are available (in Swedish) at: http://www.scb.se/vara-tjanster/bestalla-mikrodata, and requests should be emailed to mikrodata.individ@scb.se. Restrictions apply, and requests are evaluated for ethical and legal compliance with the conditions set by Statistics Sweden and the Swedish government. See http://www.scb.se/vara-tjanster/bestalla-mikrodata/utlamnande-av-mikrodata-for-forskningsandamal/ for more details.

The advantage of mobility history data is that it provides true measures of real mobility decisions. Additionally, because the data measure choices made by heterogeneous households for neighbourhoods that vary in a wide range of attributes, we can represent neighbourhood choices using a rich set of household and neighbourhood covariates [14]. The GeoSweden database is constructed from a number of different annual administrative registers. Swedish register data is a rich data source for socio-economic, geographic and demographic data analysis. It is collected by Statistics Sweden and is updated annually. The subset dataset that we used in our analysis includes the total population of the Stockholm Economic Area, which makes it possible to identify and track movers. Stockholm has relatively high incomes and housing costs, lower levels of unemployment and higher levels of job creation than the rest of Sweden. The Stockholm metropolitan area also stands out with low average ages, higher than average educational levels, greater shares of migrants born abroad and low fertility rates. The within Stockholm variation between neighbourhoods in demographic structure, socio-economic status and housing market characteristics is considerable, making Stockholm a good candidate for studies of neighbourhood choices [48]. The GeoSweden data are particularly suitable for constructing neighbourhood histories because there is almost no attrition (as it is based on register data), and as a result we were able to construct neighbourhood histories for the full population of home leavers in the Stockholm metropolitan region. This would not have been possible using panel data, which often exhibit a high rate of participant attrition in the first years of data collection. This attrition results in a high number of incomplete neighbourhood histories.

Our neighbourhoods are defined as SAMS (Small Area Market Statistics) areas. The SAMS area division is made by Statistics Sweden in collaboration with each municipality and is based on homogeneity in function. It is an often used proxy for neighbourhoods (e.g. [49–51]). The Stockholm SAMS areas have between 0 and 20,000 inhabitants, with a mean of 2,179. We have removed all SAMS areas with fewer than 50 inhabitants (mostly commercial or industrial areas), 54 in total, from the analysis, together with all moves into such areas. In addition to SAMS areas we have access to 100m^2^ grid coordinates. These are used to identify movers. A move is hence defined as a change of coordinates between two points in time. The two years of data therefore correspond to one year of moving data and provides a total of 71864 household moves. After filtering out instances where either the 2006 or 2008 SAMS area was not available we were left with 56759 distinct household moves. Excluded data are the result of households which moved out of the Stockholm urban area. In these cases we do not have information about the characteristics of the possible neighbourhoods choices that might be considered. This study thus focuses exclusively on patterns of intra-regional household movements within the Stockholm area.

We focus on modelling the choices of those households who moved neighbourhood within this period, while neighbourhood characteristics are calculated from all residents present in 2006. Although we typically discuss the choices of individuals, in practice the choosers may be individuals, families, households, or other decision makers. For this study we have aggregated the individual level data to a household level. On the household level the following variables were included: dichotomous variable indicating whether household members are all Swedish born or if any household members are foreign-born, categorical variable representing three different age categories (20-30, 30-60, 60+, measured by the eldest member of the household), dichotomous variable indicating whether household members have any children and household disposable income. Age categories were chosen to represent three potentially distinct ‘life-stages’ of adult life: young household development, established middle-age and retirement. The boundaries between these were chosen by us and are not fundamental features of the original database, wherein ages are given to the nearest year. Neighbourhood level variables are the proportion of non-western born in a neighbourhood, proportion of household with children under 18 and median neighbourhood income, which is included as a continuous variable. Age distribution on the neighbourhood level was not included. Other neighbourhood variables included are number of housing units per neighbourhood and distance of the new neighbourhood from the left neighbourhood (see also Table 1). These neighbourhood characteristics were chosen as being potentially salient factors that were derivable from our database of households. Other factors, such as social connections or neighbourhood infrastructure and amenities are not included in this analysis but could in principle be added to the neighbourhood characteristic set. An additional possibility for further analysis would be to include similarity to the focal household’s existing neighbourhood through measures such as difference in income levels between the current and putative neighbourhoods.

**Table 1:** Individual and neighbourhood characteristics contributing to the utility function in this study. Each neighbourhood characteristic interacts with all individual characteristics via Eq 18.

Summary of individual and neighbourhood characteristics
**Individual** Income, *X*_*I*_ Ethnicity (All Native Swedish or Not Native), *X*_*E*_ Has children? (dichotomous variable), *X*_*C*_ Age category (20-30, 30-60, 60+), *X*_*A*_ **Neighbourhood** Number of housing units, *Z*_*N*_ Distance from current neighbourhood, *Z*_*D*_ Mean income of residents, *Z*_*I*_ Proportion of non-western residents, *Z*_*E*_ Proportion of residents with children, *Z*_*C*_

### 2.1 Model implementation

We build a non-parametric Gaussian process (GP) model that assumes an additive structure for each neighbourhood characteristic, based on the individual characteristics. This additive structure is not a necessary condition for implementing such a GP model, but serves the purpose of clearly separating the effects of different neighbourhood factors and their interaction with individual characteristics, and makes visualising the results more straightforward. The number of housing units simply corrects the probability of choosing between unequally sized neighbourhoods. All other effects are modelled as functions drawn from Gaussian processes:

$$\begin{array}{c} \begin{array}{cc} U= & \text{log}\;Z_{N}+f_{D}\left(X_{I},X_{E},X_{C},X_{A},Z_{D}\right)+f_{I}\left(X_{I},X_{E},X_{C},X_{A},Z_{I}\right)\\ & +f_{E}\left(X_{I},X_{E},X_{C},X_{A},Z_{E}\right)+f_{C}\left(X_{I},X_{E},X_{C},X_{A},Z_{C}\right) \end{array} \end{array}$$ (18)

where *f*_*D*_, *f*_*E*_, *f*_*I*_ and *f*_*C*_ are the functions representing the contribution of neighbourhood distance, ethnicity, income and proportion of families with children respectively, with different hyper parameters and therefore covariance functions for each effect. The prior distributions over these functions are therefore specified as four independent Gaussian processes:

$$\begin{array}{c} f_{D}∼GP(0,k_{d}\left(\cdot ,\cdot \right)),\\ f_{E}∼GP(0,k_{e}\left(\cdot ,\cdot \right)),\\ f_{I}∼GP(0,k_{i}\left(\cdot ,\cdot \right)),\\ f_{C}∼GP(0,k_{c}\left(\cdot ,\cdot \right)). \end{array}$$ (19)

We follow the learning procedures specified above to infer the posterior distribution over these four functions. For each household move we compare the chosen neighbourhood with 99 other randomly chosen neighbourhoods, reweighting the utility of each neighbourhood by its selection probability under this random sampling, as specified by [14]. For the purposes of future development of this methodology it should be noted that reducing the effective size of the choice set through dimensionality-reduction techniques such as random projections [52] may offer greater accuracy than this procedure. However, here we have continued with the standard technique of random choice sampling.

## 3 Results

In this section we describe our analysis of the Stockholm residential data, using the previously described model implementation. We first give the results of feature selection, performed by Bayesian model comparison, to determine neighbourhood characteristics that are relevant in predicting household neighbourhood choice. We then look in more detail at the precise form of the inferred utility functions that govern these choices.

### 3.1 Feature selection and model comparison

**Fig 1:**
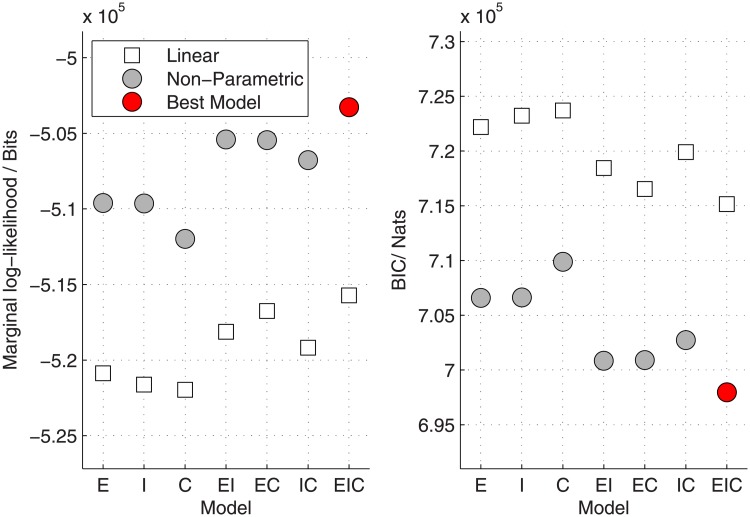
Feature selection and model comparison, showing the marginal log-likelihoods (left panel) and BIC values (right panel) for linear and non-parametric neighbourhood choice models with all possible sets of predictive features. Log-likelihoods are evaluated in base 2 and thus shown in units of bits, while BIC is evaluated in the natural logarithmic base and thus reported in units of nats. This analysis shows that the ethnic composition (E), average income (I), and proportion of households with children (C) in a neighbourhood are all relevant in neighbourhood selection, and that neighbourhood choices are far better predicted by a flexible non-parametric model than a traditional linear model.

We perform feature selection to determine which neighbourhood characteristics households use in their decision-making process. We used the marginal likelihood of the data to select which features were the best predictors of neighbourhood choices, and whether a traditional linear model or a non-parametric model best fitted the structure of neighbourhood preferences. Fig 1 shows the marginal log-likelihoods and BIC values for linear and non-parametric models with different sets of predictive neighbourhood features. We include distance to the target neighbourhood in all models for this analysis. Further analysis investigating the impact of excluding distance to the target neighbourhood shows that this is severely detrimental (see S1 Fig). Our comparison shows that a non-parametric model fits the data substantially better than a linear model across all possible combinations of neighbourhood characteristics, as indicated by both the values of the marginal log-likelihood and the BIC. As an aid to understanding the scale of this difference, each observed move was 1.16 times as probable conditioned on the best non-parametric model as it was conditioned on the best linear model, based on the geometric mean of the marginal likelihoods. In addition to distance from the current neighbourhood, all three further neighbourhood characteristics are selected in the best performing model (indicated in red), showing that households choosing a new abode take neighbourhood ethnicity, income and proportion of households with children into account when making their decision.

#### 3.1.1 Inferred utility function

**Fig 2:**
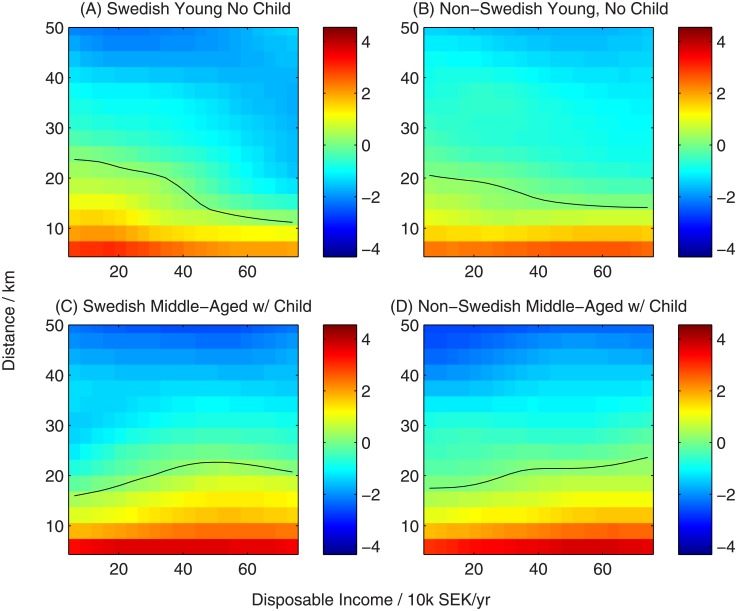
Utility function for neighbourhood distance from the household’s current abode. Black contours represent a utility of zero, indicating no overall preference for or against neighbourhoods with this characteristic. Candidate neighbourhood distances extend to 50 kilometres, within the Stockholm urban area.

**Fig 3:**
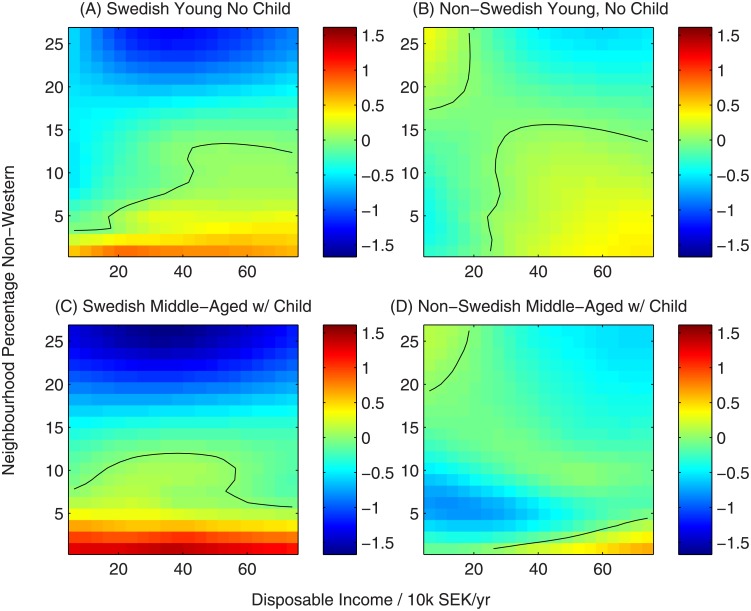
Utility function for neighbourhood share of non-western residents. Black contours represent a utility of zero, indicating no overall preference for or against neighbourhoods with this characteristic.

**Fig 4:**
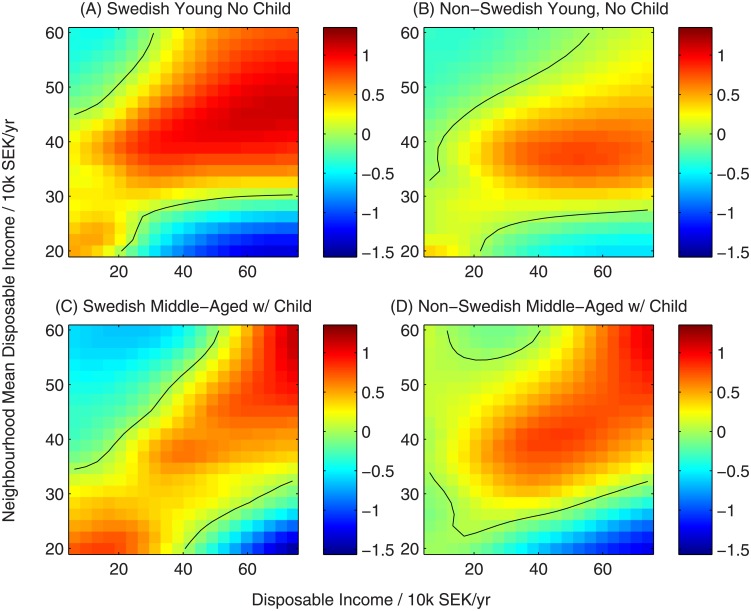
Utility function for neighbourhood income. Black contours represent a utility of zero, indicating no overall preference for or against neighbourhoods with this characteristic.

**Fig 5:**
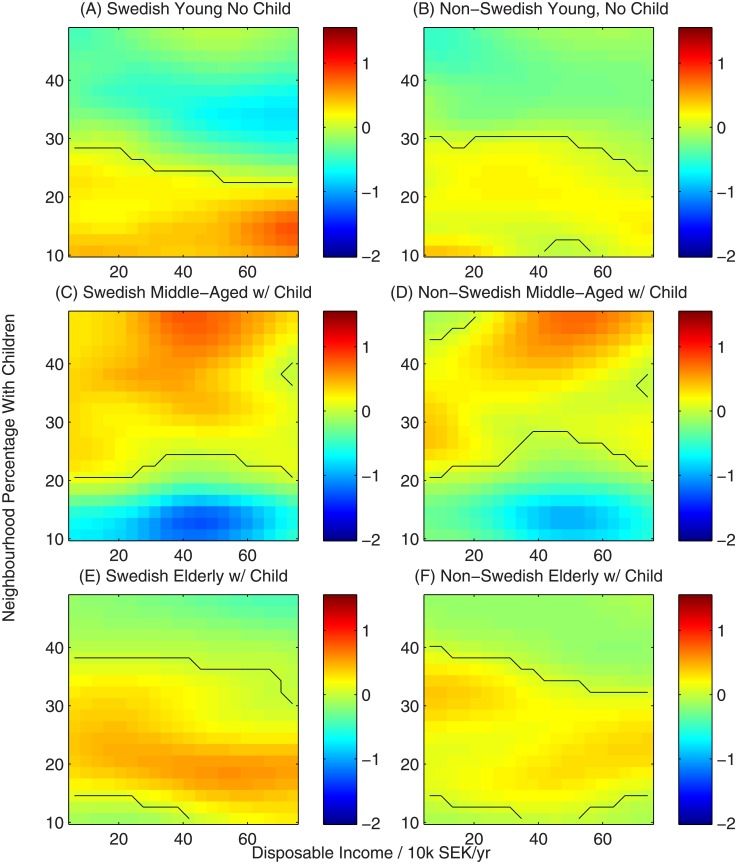
Utility function for neighbourhood percentage of residents with children. Black contours represent a utility of zero, indicating no overall preference for or against neighbourhoods with this characteristic.

In Figs 2–5 we show a variety of perspectives of the inferred function linking household characteristics, neighbourhood characteristics and utility. In each figure the element of the utility function corresponding to a specific neighbourhood characteristic is shown as a function of household income for all-native Swedish households and non-Swedish households across two sub-partitions: young households (below 30 years old) without children and middle-aged households (30-60 years) with children to demonstrate their contrasting preferences. The colour scale represents the value of the utility function, indicating the strength of preference. Black lines show contours of zero utility of zero, indicating no preference for or against the choice.

A note on interpreting the magnitude of utility functions: the effect of any change in utility for one option in terms of the probability of selection will vary, depending on the original utility of the focal option and the utilities of competing options, through the effect of Eq 3. An approximate effect size translation can be given by considering the case in which both the focal option and all competing options have utility zero before considering the additional utility of interest. In this case the probability of selecting the focal option with additional utility *U* will change from 1/*N* to approximately exp(*U*)/*N*, where *N* is the number of possible choice options, indicating a exp(*U*)-fold increase in probability.

Utility decreases with distance from the current neighbourhood (Fig 2). Households of all ethnicities, ages and incomes show a strong preference for neighbourhoods closer to their current abode. The pattern of preference for local neighbourhoods is generally very consistent across all ages groups, incomes and ethnicities, though there is a weak trend for young, childless households to be more accepting of more distant moves when poorer, and the reverse trend for middle-aged households with children, regardless of ethnicity. By absolute magnitude of the utility this is the strongest effect when choosing a new neighbourhood for all households categories.

Response to neighbourhood immigrant populations shows a marked difference based on the household’s own ethnicity (Fig 3). All-native households, both without (A) and with (C) children show a consistent preference for neighbourhoods with low non-western populations. In contrast, the preferences of non-Swedish households are income dependent: non-Swedish households with low incomes preferentially choose neighbourhoods with a high non-western population, while the preference of high-income households follows the direction of all-native households (albeit less strongly). The diagonal banding in panels B and D, showing non-monotonic neighbourhood diversity preferences for non-Swedish households with intermediate incomes, is a feature that a linear model could not capture.

The utility of a neighbourhood based on the income of its residents shows strong signs of homophily (Fig 4). Households tend to prefer neighbourhoods where incomes match or slightly exceed their own. Because people’s ability to move to a neighbourhood is limited by whether they can afford to buy or rent a housing unit, this does not strictly specify a preference for neighbourhoods of similar incomes, but rather an increased tendency to move to these areas—potentially as households preferentially choose the wealthiest neighbourhoods they can afford. The utility functions shown here are a good demonstration of non-linearity—for intermediate household incomes the utility of a neighbourhood increases with neighbourhood income up to a point slightly above the household income, then declines. This non-monotonic preference, for neighbourhood incomes neither too high nor too low, would not be detected by a linear model unless the neighbourhood income variable was transformed before analysis (for example, using a transformation of absolute difference between neighbourhood mean income and ego’s income).

The response to the percentage of households in a neighbourhood with children also displays strong homophily (Fig 5). Childless households universally prefer neighbourhoods with a low percentage of parents, while those with children prefer neighbourhoods with a high percentage of parents. Preferences for all four groups in panels A-D are broadly monotonic in the proportion of households with children. A partial exception to these homophilic preferences is seen in the choices made by those in the oldest age category (60+, panels E-F). In this case those with children display preferences that lie between those of middle-aged households with children and those of young households without children, showing a tendency to choose neighbourhoods with an intermediate percentage of households with children.

## 4 Discussion

Using a large data set of household moves, we demonstrated that a non-parametric version of the conditional logit model provides a substantially better description of neighbourhood choices than a standard linear implementation. The non-parametric model both provides a more accurate fit to the data, and therefore better predictive potential, but also allows us to infer complex household preferences that would not be obtainable with a linear model. Our model selection using the two model types revealed that all three neighbourhood characteristics tested (ethnicity, income and proportion of households with children) contributed significantly to households neighbourhood choice.

Our results indicate a strong degree of homophily in the selection of a new neighbourhood for moving households, but with significant variations from the general pattern for specific groups based on the household factors of age, ethnicity, income and number of children. Notably, non-native households with high income preferred neighbourhoods with a low percentage of non-western residents. This is closer to to the preference of all-native households, following the same directionality but more weakly. While in general households with children preferred neighbourhoods with a relatively high proportion of parents, those in the highest age group displayed a more intermediate preference for areas with neither very high nor very low numbers of children. This may reflect the fact that for many their children have left home, or a change in priorities when children are older. Whether homophilic preferences are driven by a genuine preference amongst those with children for the presence of other families with children, or whether this reflects a shared attraction amongst such families for features such as play areas and schools is not discernible from this analysis alone. However, common sense would dictate that common attraction to amenities is at least partly responsible. While all households showed a preference for neighbourhoods with a mean income either matching or slightly exceeding their own (which probably reflects being priced out of more expensive neighbourhoods), this preference was far stronger for the households with the highest incomes.

All households strongly preferred to move to neighbourhoods close to their current abode. This may reflect both social ties to the local area (we did not have access to data on individuals’ social networks), persistence of place of employment anchoring the home location and/or simply an availability bias where the individual is more aware of available abodes in the local vicinity. With more detailed data on social ties, place of employment and potentially even recorded movements (for example via GPS enabled smartphones) these effects could be investigated further in subsequent work.

A detailed analysis of the motivating factors for neighbourhood selection such as this potentially provides the platform for an agent-based simulation model of household movements in a city, with agents choosing their new neighbourhoods based on the inferred utility functions and the current status of the other agents in the simulation. Such a model could provide the linkage between this analysis of household choices and an explanation of the observed macro-scale patterns of segregation and neighbourhood characteristics. Combined with models of demographic development and change to predict households income and family status over time, this could lead to a realistic forecasting model for residential patterns in the future.

As very large data sets of human activity become the norm, the potential exists to explore in far greater detail than before exactly what motivates the choices of individuals, families and societies. Identifying the precise mechanisms behind individual or household choices is a keystone in the process of analytical sociology, which aims to explore macro-level societal facts and their relationships by linkage to the micro-level decisions and motivations of individuals [53]. However, precise evaluation of these motivations from choice data poses many challenges. In particular, with many possible covarying factors influencing each decision, accurately controlling for each factor is crucial. For example, if income and ethnicity covary, an overly simple (e.g. linear) controlling regression for income may lead to apparent effects of ethnicity that are in fact artefacts of the more complex role of income. Our results showed that the best model including two neighbourhood factors from ethnicity, income and number of children was disjoint from the best one factor model, selecting ethnicity and number of children as opposed to income. This also varied between the non-parametric and the linear models, showing not only that relevant controls are important to identify the real effect a factor has, but also that using a standard linear variable will not suffice to control for the influence of a factor with a complex influence.

The use of Gaussian processes in an extended conditional logit model offers a solution to this problem, since the dependence of the utility function on each factor can be almost arbitrarily complex, with the degree of complexity driven by the data available. However, by providing a prior distribution over functions that naturally favours smoother, less complex forms, the Gaussian process framework also provides a natural barrier to overfitting. The data must show convincing evidence for a complex utility function before it will be selected. In combination with flexible ARD covariance functions, we identify complex relations between the utility and highly relevant household and neighbourhood characteristics, while Bayesian model selection is used to prevent the identification of spurious relationships with irrelevant factors.

As with any study using observational data, causality is difficult to pin down precisely using this method. This is especially true with homophilic effects: do households choose neighbourhoods because of their high percentage of ethnically similar households, or is there an unseen aspect of a neighbourhood that makes it persistently attractive to those of a particular ethnicity, thus creating the illusion of homophily? Controlling as accurately as possible for other observable factors, and looking for consistent effect sizes across studies can make the inference of homophily stronger but it is likely impossible to be sure in any single observational study that such an effect is truly causal. Studies such as this should be seen as identifying persistent patterns in the data worthy of theoretical and further empirical investigation.

In common with other regression studies there is an asymmetry between positive and negative findings. While an inferred utility function with a large effect size is clear evidence for a real underlying factor influencing decisions, a negative finding does not necessarily imply the absence of such a factor—it may simply indicate a lack of relevant data. Whereas in the classic conditional logit model, a negative finding would be revealed by a non-significant regression coefficient, in this non-parametric model the utility function itself is not significantly different from zero in some regions. The Gaussian process prior returns the utility function to zero where relevant data is sparse. Our study has more data points associated with Swedish households than non-Swedish, and more data from middle-income households than extreme incomes. Therefore weak effect sizes in extreme income and/or non-Swedish households should be treated with caution, since the available data may not have the power to reveal an existing decision factor.

Compared to a classic parametric regression approach, the inferred utility functions from our analysis are less easily summarised by a concise set of regression coefficients. Readers familiar with regression models may expect a table of significant and non-significant coefficients, or a forest plot, to assess which factors are relevant. However, this limitation is in fact a feature, not a bug of the method. The traditional model, being overly structured, provides superficial simplicity while offering a false confidence that different effects are being adequately controlled for. A large degree of user ‘tweaking’ is required (e.g. through the explicit inclusion of non-linear basis functions) to model complex data and as such the temptation is to test many different possible interaction effects and basis functions until a significant finding is achieved. With our model the researcher should study and report the inferred utility functions directly, rather than through summary statistics. Questions such as ‘does factor X influence neighbourhood choice’ are answerable through model selection as per Fig 1, and the confidence in those judgements is obtained by evaluating the Bayes factor [40], the ratio of the marginal likelihoods for a model including X and an equivalent model excluding X.

## Supporting information

S1 Fig Model comparison with and without distance to candidate neighbourhood. Marginal log-likelihoods (left panel) and BIC values (right panel) are shown for non-parametric models using different combinations of predictive features, including or excluding distance to the candidate neighbourhood as an additional feature.
(EPS)
